# Supplementary material for: An Association Study of HLA with the Kinetics of SARS-CoV-2 Spike Specific IgG Antibody Responses to BNT162b2 mRNA Vaccine
Source: Vaccines (Basel). 2022 Apr 5;10(4):563. doi: 10.3390/vaccines10040563 (PMC9029840; doi:10.3390/vaccines10040563)
Supplement: Supplementary file 1 [file vaccines-10-00563-s001.zip › Supplementary Figures.pdf]

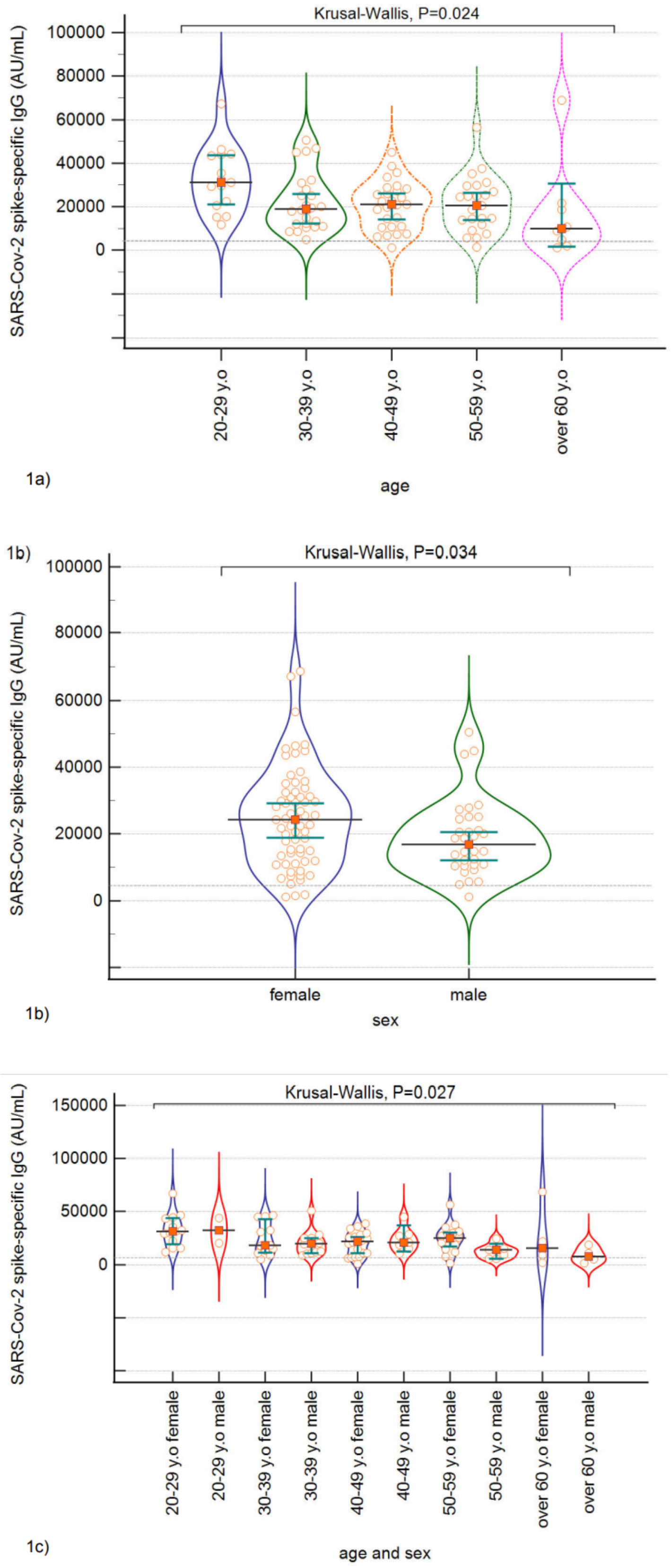

Supplementary Figure S1. SARS-CoV-2 spike-specific IgG production on day 29, stratified by age and sex (**a**) SARS-CoV-2 spike-specific IgG production on day 29, stratified by 5 age groups: 20–29 years old (y.o), 30–39 y.o., 40–49 y.o. 50–59 y.o, and over 60 y.o. (**b**) SARS-CoV-2 spike-specific IgG

production on day 29, stratified by sex. (**c**) SARS-CoV-2 spike-specific IgG production on day 29, stratified by 10 groups including the categories listed in (**a**) and (**b**) Violin plots showing median and 95% confidence interval (CI), grey dotted line indicates IgG-S level of 4160 AU/mL, which corresponded to a 95% probability of obtaining a PRNT ID50 (estimated number of virus particles required to produce infection in 50% of normal adult humans) at 1:250 dilution .

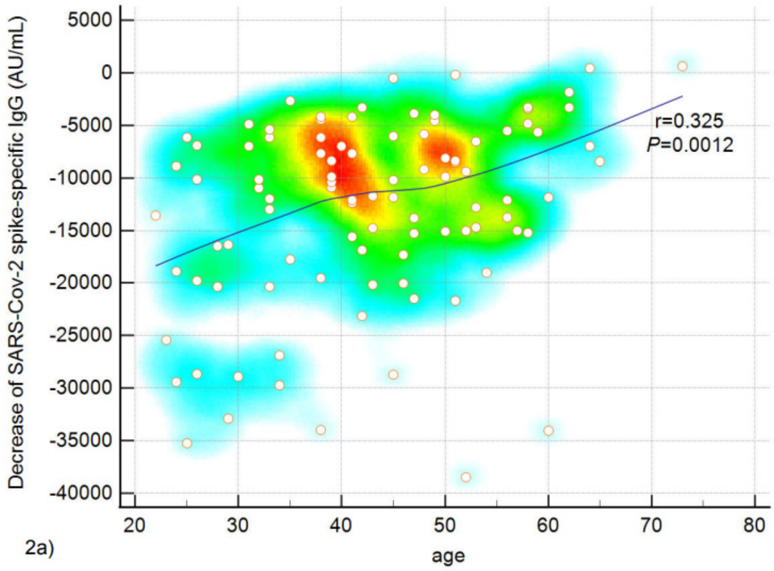

2a)

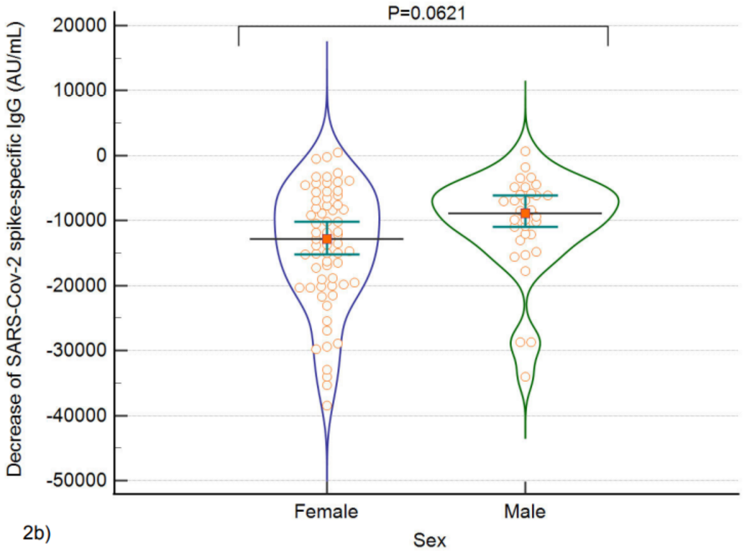

2b)

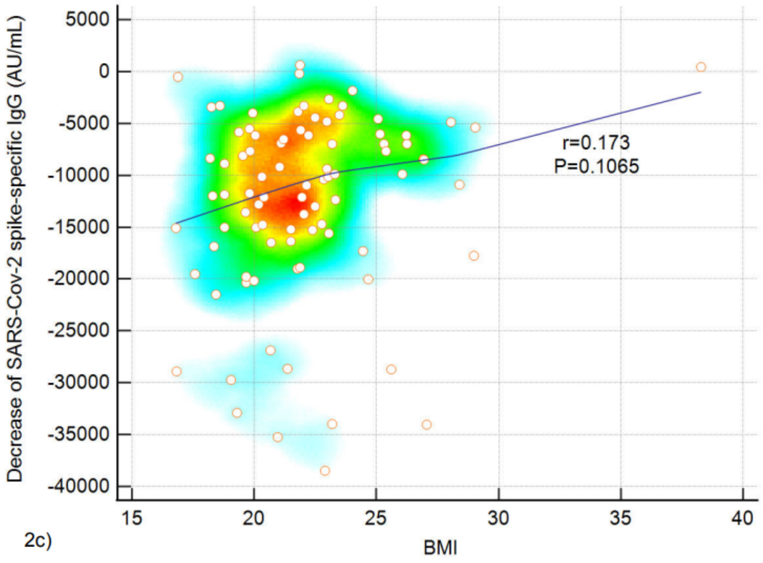

2c)

Supplementary Figure S2. SARS-CoV-2 spike-specific IgG decline on day 61, stratified by age, sex, and BMI of participants (**a**) Scatter plot showing the correlation between age and the change in IgG-S between day 29 and day 61. Correlation coefficients and P-values are calculated. Color intensity of the heatmap indicates the accumulation of data points in a specific area. (**b**) Violin plot showing the correlation between sex and the change in IgG-S between day 29 and day 61. Violin plots showing median and 95% confidence interval (CI), grey dotted line indicates IgG-S level of 4160 AU/mL, which corresponded to a 95% probability of obtaining a PRNT ID50 (estimated number of virus particles required to produce infection in 50% of normal adult humans) at 1:250 dilution (**c**) Scatter plot showing the correlation between the BMI of participants and the change in IgG-S between day 29 and day 61. Correlation coefficients and p-values are calculated. Color intensity of the heatmap indicates the accumulation of data points in a specific area.

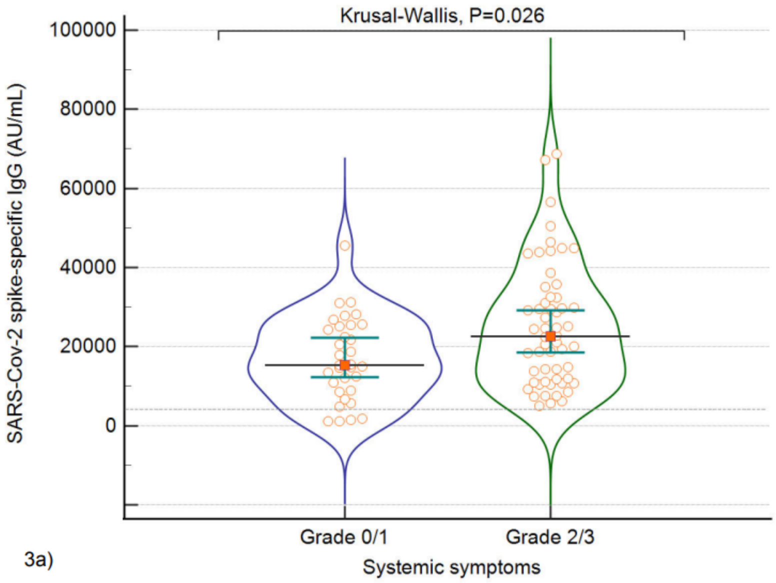

3a)

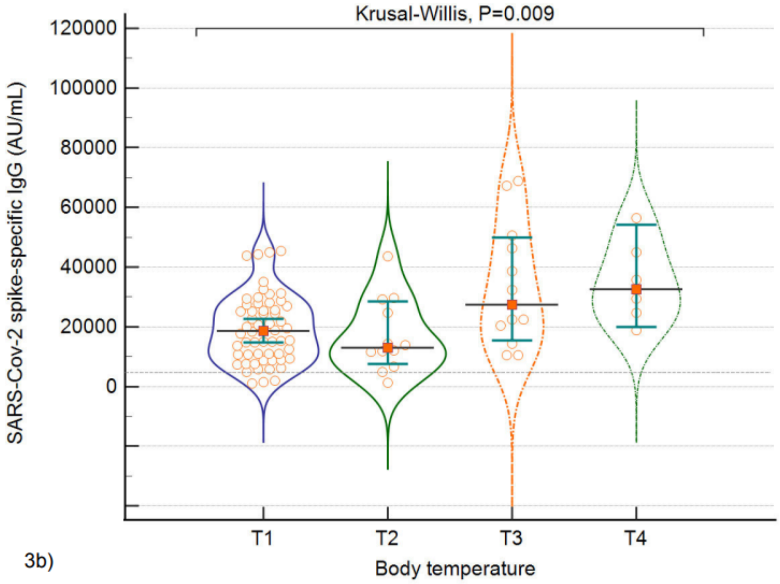

3b)

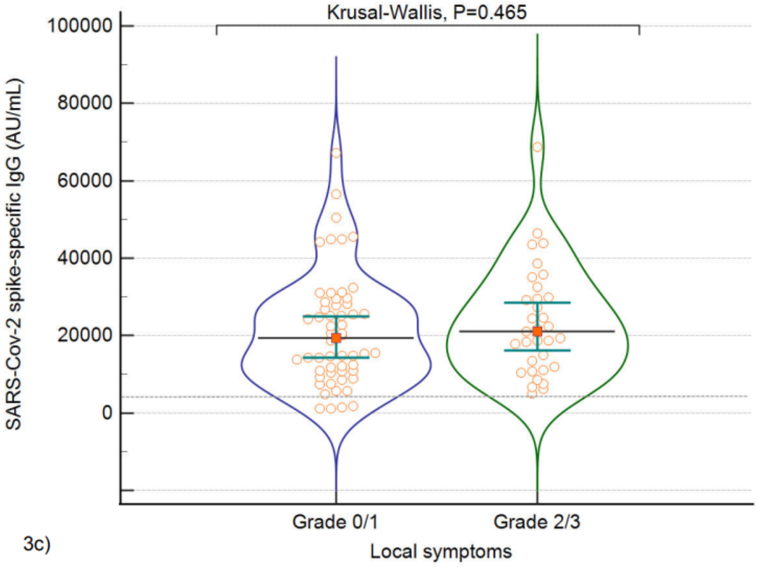

3c)

Supplementary Figure S3. Association between SARS-CoV-2 spike-specific IgG production on day 29 and adverse reactions after two doses of BNT162b2 vaccination (**a**) Violin plot showing the distribution of IgG-S production on day 29 stratified by grades of systemic symptoms experienced by participants after two doses of BNT162b2 vaccination (**b**) Violin plot showing the distribution of IgG-S production on day 29 stratified by fever experienced by participants after two doses of BNT162b2 vaccination. T1: < 37.5°C; T2: 37.5–37.9 °C; T3: 38.0–38.4 °C; and T4: 38.5–38.9 °C. (**c**) Violin plot showing the distribution of IgG-S production on day 29 stratified by the grade of local symptoms experienced by participants after two doses of BNT162b2 vaccination Violin plots showing median and 95% confidence interval (CI), grey dotted line indicates IgG-S level of 4160 AU/mL, which corresponded to a 95% probability of obtaining a PRNT ID50 (estimated number of virus particles required to produce infection in 50% of normal adult humans) at 1:250 dilution
