# Supplementary material for: An Association Study of HLA with the Kinetics of SARS-CoV-2 Spike Specific IgG Antibody Responses to BNT162b2 mRNA Vaccine
Source: Vaccines (Basel). 2022 Apr 5;10(4):563. doi: 10.3390/vaccines10040563 (PMC9029840; doi:10.3390/vaccines10040563)
Supplement: Supplementary file 1 [file vaccines-10-00563-s001.zip › Supplementary Tables.pdf]

Supplementary Table S1 | Associational analysis of HLA-A, -C, -B, -DRB1, -DQB1, -DOA1 and -DPB1 alleles in 55 participants with no local adverse effect versus 33 participants with local adverse effect

Locus	Two-field allele	No adverse effect (2n=110) %	Adverse effect (2n=66) %	OR (95%CI)	P-value	Three-field allele	No adverse effect (2n=110) %	Adverse effect (2n=66) %	OR (95%CI)	P-value	Four-field allele	No adverse effect (2n=110) %	Adverse effect (2n=66) %	OR (95%CI)	P-value
A	02:06	15.5	10.6	0.65 (0.21-1.77)	0.364	02:06:01	15.5	10.6	0.65 (0.21-1.77)	0.364	02:06:01:01	11.8	6.1	0.48 (0.11-1.66)	0.211
A	11:01	10.9	10.6	0.97 (0.31-2.85)	0.950	11:01:01	10.9	10.6	0.97 (0.31-2.85)	0.950	11:01:01:01	10.9	10.6	0.97 (0.31-2.85)	0.950
A	24:02	34.5	34.8	1.01 (0.50-2.01)	0.967	24:02:01	34.5	34.8	1.01 (0.50-2.01)	0.967	24:02:01:01	32.7	33.3	1.03 (0.51-2.06)	0.934
A	31:01	7.3	10.6	1.51 (0.44-5.03)	0.443	31:01:02	7.3	10.6	1.51 (0.44-5.03)	0.443	31:01:02:01	6.4	10.6	1.75 (0.49-6.13)	0.314
A	binned	31.8	33.3	1.07 (0.53-2.15)	0.835	binned	31.8	33.3	1.07 (0.53-2.15)	0.835	binned	38.2	39.4	1.05 (0.53-2.06)	0.873
C	01:02	16.4	16.7	1.02 (0.40-2.48)	0.958	01:02:01	16.4	16.7	1.02 (0.40-2.48)	0.958	01:02:01:01	12.7	7.6	0.56 (0.15-1.76)	0.286
C	03:03	9.1	15.2	1.79 (0.62-5.09)	0.220	03:03:01	9.1	15.2	1.79 (0.62-5.09)	0.220	03:03:01:01	9.1	15.2	1.79 (0.62-5.09)	0.220
C	03:04	12.7	6.1	0.44 (0.10-1.50)	0.158	03:04:01	12.7	6.1	0.44 (0.10-1.50)	0.158	03:04:01:02	12.7	6.1	0.44 (0.10-1.50)	0.158
C	07:02	10.9	9.1	0.82 (0.24-2.50)	0.700	07:02:01	10.9	9.1	0.82 (0.24-2.50)	0.700					
C	08:01	8.2	9.1	1.12 (0.31-3.73)	0.834	08:01:01	8.2	9.1	1.12 (0.31-3.73)	0.834	08:01:01:01	8.2	9.1	1.12 (0.31-3.73)	0.834
C	12:02	11.8	15.2	1.33 (0.49-3.53)	0.525	12:02:02	10.9	15.2	1.46 (0.53-3.95)	0.410	12:02:02:01	10.9	15.2	1.46 (0.53-3.95)	0.410
C	binned	30.9	28.8	0.90 (0.43-1.85)	0.766	binned	31.8	28.8	0.87 (0.42-1.77)	0.673	binned	46.4	47.0	1.02 (0.53-1.98)	0.938
B	35:01	9.1	10.6	1.19 (0.36-3.67)	0.742	35:01:01	9.1	10.6	1.19 (0.36-3.67)	0.742					
B	40:02	11.8	6.1	0.48 (0.11-1.66)	0.211	40:02:01	11.8	6.1	0.48 (0.11-1.66)	0.211	40:02:01:01	10.0	4.5	0.43 (0.07-1.72)	0.195
B	51:01	10.0	9.1	0.90 (0.26-2.82)	0.843	51:01:01	10.0	9.1	0.90 (0.26-2.82)	0.843					
B	52:01	11.8	15.2	1.33 (0.49-3.53)	0.525	52:01:01	11.8	15.2	1.33 (0.49-3.53)	0.525	52:01:01:02	11.8	15.2	1.33 (0.49-3.53)	0.525
B	binned	57.3	59.1	1.08 (0.56-2.10)	0.813	binned	57.3	59.1	1.08 (0.56-2.1)	0.813	binned	78.2	80.3	1.14 (0.51-2.65)	0.738
DRB1	04:05	10.0	10.6	1.07 (0.33-3.21)	0.898	04:05:01	10.0	10.6	1.07 (0.33-3.21)	0.898	04:05:01:01	10.0	10.6	1.07 (0.33-3.21)	0.898
DRB1	09:01	16.4	6.1	0.33 (0.08-1.07)	0.045	09:01:02	16.4	6.1	0.33 (0.08-1.07)	0.045	09:01:02:01	16.4	6.1	0.33 (0.08-1.07)	0.045
DRB1	15:01	9.1	12.1	1.38 (0.45-4.12)	0.521	15:01:01	9.1	12.1	1.38 (0.45-4.12)	0.521	15:01:01:01	9.1	10.6	1.19 (0.36-3.67)	0.742
DRB1	15:02	10.0	10.6	1.07 (0.33-3.21)	0.898	15:02:01	10.0	10.6	1.07 (0.33-3.21)	0.898	15:02:01:01	10.0	10.6	1.07 (0.33-3.21)	0.898
DRB1	binned	54.5	60.6	1.28 (0.66-2.51)	0.432	binned	54.5	60.6	1.28 (0.66-2.51)	0.432	binned	54.5	62.1	1.37 (0.70-2.68)	0.325
DQA1	01:02	13.6	15.2	1.13 (0.42-2.90)	0.780	01:02:01	13.6	13.6	1.00 (0.36-2.63)	1.000	01:02:01:01	9.1	10.6	1.19 (0.36-3.67)	0.742
DQA1	01:03	18.2	18.2	1.00 (0.41-2.35)	1.000	01:03:01	18.2	18.2	1.00 (0.41-2.35)	1.000	01:03:01:01	10.0	13.6	1.42 (0.49-4.02)	0.462
DQA1	01:04	10.0	4.5	0.43 (0.07-1.72)	0.195	01:04:01	10.0	4.5	0.43 (0.07-1.72)	0.195	01:04:01:01	10.0	4.5	0.43 (0.07-1.72)	0.195
DQA1	03:01	11.8	13.6	1.18 (0.42-3.19)	0.724	03:01:01	11.8	13.6	1.18 (0.42-3.19)	0.724	03:01:01:01	11.8	13.6	1.18 (0.42-3.19)	0.724
DQA1	03:02	18.2	7.6	0.37 (0.10-1.09)	0.051	03:02:01	18.2	7.6	0.37 (0.10-1.09)	0.051	03:02:01:01	17.3	7.6	0.39 (0.11-1.17)	0.070
DQA1	03:03	13.6	12.1	0.87 (0.30-2.36)	0.773	03:03:01	13.6	12.1	0.87 (0.30-2.36)	0.773	03:03:01:03	9.1	10.6	1.19 (0.36-3.67)	0.742
DQA1	binned	14.5	28.8	2.38 (1.05-5.41)	0.022	binned	14.5	30.3	2.55 (1.13-5.79)	0.012	binned	32.7	39.4	1.34 (0.67-2.64)	0.370
DQB1	03:01	9.1	15.2	1.79 (0.62-5.09)	0.220	03:01:01	9.1	15.2	1.79 (0.62-5.09)	0.220					
DQB1	03:02	11.8	13.6	1.18 (0.42-3.19)	0.724	03:02:01	11.8	13.6	1.18 (0.42-3.19)	0.724	03:02:01:01	11.8	13.6	1.18 (0.42-3.19)	0.724
DQB1	03:03	17.3	7.6	0.39 (0.11-1.17)	0.070	03:03:02	17.3	7.6	0.39 (0.11-1.17)	0.070	03:03:02:02	17.3	7.6	0.39 (0.11-1.17)	0.070
DQB1	04:01	9.1	10.6	1.19 (0.36-3.67)	0.742	04:01:01	9.1	10.6	1.19 (0.36-3.67)	0.742	04:01:01:01	9.1	10.6	1.19 (0.36-3.67)	0.742
DQB1	06:01	18.2	16.7	0.90 (0.36-2.15)	0.798	06:01:01	18.2	16.7	0.90 (0.36-2.15)	0.798	06:01:01:01	18.2	16.7	0.90 (0.36-2.15)	0.798
DQB1	06:02	9.1	10.6	1.19 (0.36-3.67)	0.742	06:02:01	9.1	10.6	1.19 (0.36-3.67)	0.742	06:02:01:01	9.1	10.6	1.19 (0.36-3.67)	0.742
DQB1	binned	25.5	25.8	1.02 (0.47-2.15)	0.964	binned	25.5	25.8	1.02 (0.47-2.15)	0.964	binned	34.5	40.9	1.31 (0.66-2.57)	0.397
DPA1	01:03	43.6	40.9	0.89 (0.46-1.73)	0.723	01:03:01	43.6	40.9	0.89 (0.46-1.73)	0.723	01:03:01:01	30.9	22.7	0.66 (0.30-1.39)	0.241
DPA1											01:03:01:05	9.1	13.6	1.58 (0.53-4.60)	0.347
DPA1	02:01	14.5	21.2	1.58 (0.66-3.76)	0.255	02:01:01	14.5	21.2	1.58 (0.66-3.76)	0.255	02:01:01:02	9.1	19.7	2.45 (0.92-6.67)	0.043
DPA1	02:02	41.8	37.9	0.85 (0.43-1.66)	0.606	02:02:02	41.8	37.9	0.85 (0.43-1.66)	0.606	02:02:02:01	41.8	37.9	0.85 (0.43-1.66)	0.606
DPA1											binned	9.1	6.1	0.65 (0.14-2.36)	0.472
DPB1	02:01	26.4	18.2	0.62 (0.27-1.39)	0.214	02:01:02	26.4	18.2	0.62 (0.27-1.39)	0.214	02:01:02:01	20.9	15.2	0.68 (0.27-1.61)	0.343
DPB1	04:02	9.1	13.6	1.58 (0.53-4.60)	0.347	04:02:01	9.1	13.6	1.58 (0.53-4.60)	0.347	04:02:01:02	9.1	12.1	1.38 (0.45-4.12)	0.521
DPB1	05:01	36.4	37.9	1.07 (0.54-2.10)	0.840	05:01:01	36.4	37.9	1.07 (0.54-2.10)	0.840	05:01:01:01	31.8	28.8	0.87 (0.42-1.77)	0.673
DPB1	09:01	7.3	15.2	2.28 (0.76-7.02)	0.095	09:01:01	7.3	15.2	2.28 (0.76-7.02)	0.095	09:01:01	7.3	15.2	2.28 (0.76-7.02)	0.095
DPB1	binned	20.9	15.2	0.68 (0.27-1.61)	0.343	binned	20.9	15.2	0.68 (0.27-1.61)	0.343	binned	30.9	28.8	0.90 (0.43-1.85)	0.766

Supplementary Table S2 | Associational analysis of HLA-A, -C, -B, -DRB1, -DQB1, -DOA1 and -DPB1 alleles in 70 participants with fever versus 18 participants with fever more than 38°C

Locus	Two-field allele	No fever (2n=140) %	Fever (>38°C) (2n=36) %	OR (95%CI)	p-value	Three-field allele	No fever (2n=140) %	Fever (>38°C) (2n=36) %	OR (95%CI)	p-value	Four-field allele	No fever (2n=140) %	Fever (>38°C) (2n=36) %	OR (95%CI)	p-value
A	02:06	13.6	16.7	1.27 (0.38-3.69)	0.635	02:06:01	13.6	16.7	1.27 (0.38-3.69)	0.635					
A	24:02	36.4	27.8	0.67 (0.27-1.58)	0.331	24:02:01	36.4	27.8	0.67 (0.27-1.58)	0.331	24:02:01:01	33.6	27.8	0.76 (0.30-1.80)	0.508
A	binned	50.0	55.6	1.25 (0.56-2.81)	0.552	binned	50.0	55.6	1.25 (0.56-2.81)	0.552	binned	66.4	72.2	1.31 (0.55-3.31)	0.508
C	01:02	17.1	13.9	0.78 (0.22-2.32)	0.639	01:02:01	17.1	13.9	0.78 (0.22-2.32)	0.639					
C	binned	82.9	86.1	1.28 (0.43-4.65)	0.639	binned	82.9	86.1	1.28 (0.43-4.65)	0.639	binned	100.0	100.0	NA	NA
B	binned	100.0	100.0	NA	NA	binned	100.0	100.0	NA	NA	binned	100.0	100.0	NA	NA
DRB1	binned	100.0	100.0	NA	NA	binned	100.0	100.0	NA	NA	binned	100.0	100.0	NA	NA
DQA1	01:02	13.6	16.7	1.27 (0.38-3.69)	0.635										
DQA1	01:03	20.7	11.1	0.48 (0.11-1.52)	0.188	01:03:01	20.7	11.1	0.48 (0.11-1.52)	0.188					
DQA1	03:02	15.0	11.1	0.71 (0.17-2.32)	0.551	03:02:01	15.0	11.1	0.71 (0.17-2.32)	0.551					
DQA1	binned	50.7	61.1	1.53 (0.68-3.50)	0.265	binned	64.3	77.8	1.94 (0.78-5.3)	0.125	binned	100.0	100.0	NA	NA
DQB1	06:01	20.0	11.1	0.50 (0.12-1.59)	0.217	06:01:01	20.0	11.1	0.50 (0.12-1.59)	0.217	06:01:01:01	20.0	11.1	0.50 (0.12-1.59)	0.217
DQB1	binned	80.0	88.9	2.00 (0.63-8.39)	0.217	binned	80.0	88.9	2.00 (0.63-8.39)	0.217	binned	80.0	88.9	2.00 (0.63-8.39)	0.217
DPA1	01:03	42.9	38.9	0.85 (0.37-1.90)	0.667	01:03:01	42.9	38.9	0.85 (0.37-1.90)	0.667	01:03:01:01	28.6	25.0	0.83 (0.32-2.03)	0.670
DPA1	02:01	17.1	16.7	0.97 (0.30-2.72)	0.946	02:01:01	17.1	16.7	0.97 (0.30-2.72)	0.946					
DPA1	02:02	40.0	44.4	1.20 (0.53-2.67)	0.629	02:02:02	40.0	44.4	1.20 (0.53-2.67)	0.629	02:02:02:01	40.0	44.4	1.20 (0.53-2.67)	0.629
DPA1											binned	31.4	30.6	0.96 (0.39-2.24)	0.920
DPB1	02:01	24.3	19.4	0.75 (0.26-1.97)	0.540	02:01:02	24.3	19.4	0.75 (0.26-1.97)	0.540	02:01:02:01	18.6	19.4	1.06 (0.35-2.83)	0.905
DPB1	05:01	36.4	38.9	1.11 (0.48-2.50)	0.785	05:01:01	36.4	38.9	1.11 (0.48-2.50)	0.785	05:01:01:01	31.4	27.8	0.84 (0.33-1.99)	0.672
DPB1	binned	39.3	41.7	1.10 (0.48-2.47)	0.795	binned	39.3	41.7	1.10 (0.48-2.47)	0.795	binned	50.0	52.8	1.12 (0.50-2.50)	0.766

Supplementary Table S3 | Associational analysis of HLA-A, -C, -B, -DRB1, -DQB1, -DOA1 and -DPB1 alleles in 55 participants with no systemic adverse effect versus 33 participants with systemic adverse effect

Locus	Two-field allele	No adverse effect (n=64) %	With adverse effect (n=112) %	OR (95%CI)	p-value	Three-field allele	No adverse effect (n=64) %	With adverse effect (n=112) %	OR (95%CI)	p-value	Four-field allele	No adverse effect (n=64) %	With adverse effect (n=112) %	OR (95%CI)	p-value
A	02:06	15.6	12.5	0.77 (0.30-2.09)	0.561	02:06:01	15.6	12.5	0.77 (0.30-2.09)	0.561	02:06:01:01	12.5	8.0	0.61 (0.20-1.94)	0.335
A	11:01	10.9	10.7	0.98 (0.33-3.11)	0.963	11:01:01	10.9	10.7	0.98 (0.33-3.11)	0.963	11:01:01:01	10.9	10.7	0.98 (0.33-3.11)	0.963
A	24:02	35.9	33.9	0.92 (0.46-1.84)	0.788	24:02:01	35.9	33.9	0.92 (0.46-1.84)	0.788	24:02:01:01	34.4	32.1	0.90 (0.45-1.84)	0.762
A	31:01	10.9	7.1	0.63 (0.19-2.15)	0.386	31:01:02	10.9	7.1	0.63 (0.19-2.15)	0.386	31:01:02:01	9.4	7.1	0.74 (0.21-2.74)	0.599
A	binned	26.6	35.7	1.54 (0.75-3.23)	0.212	binned	26.6	35.7	1.54 (0.75-3.23)	0.212	binned	32.8	42.0	1.48 (0.74-2.98)	0.230
C	01:02	10.9	19.6	1.99 (0.76-5.86)	0.134	01:02:01	10.9	19.6	1.99 (0.76-5.86)	0.134	01:02:01:01	6.3	13.4	2.32 (0.69-10.01)	0.142
C	03:03	18.8	7.1	0.33 (0.11-0.96)	0.020	03:03:01	18.8	7.1	0.33 (0.11-0.96)	0.020	03:03:01:01	18.8	7.1	0.33 (0.11-0.96)	0.020
C	03:04	12.5	8.9	0.69 (0.23-2.13)	0.452	03:04:01	12.5	8.9	0.69 (0.23-2.13)	0.452	03:04:01:02	12.5	8.9	0.69 (0.23-2.13)	0.452
C	07:02	12.5	8.9	0.69 (0.23-2.13)	0.452	07:02:01	12.5	8.9	0.69 (0.23-2.13)	0.452					
C	08:01	6.3	9.8	1.63 (0.46-7.33)	0.414	08:01:01	6.3	9.8	1.63 (0.46-7.33)	0.414	08:01:01:01	6.3	9.8	1.63 (0.46-7.33)	0.414
**C**	**12:02**	**20.3**	**8.9**	**0.38 (0.14-1.03)**	**0.031**	**12:02:02**	**18.8**	**8.9**	**0.42 (0.15-1.16)**	**0.058**	**12:02:02:01**	**18.8**	**8.9**	**0.42 (0.15-1.16)**	**0.058**
C	binned	18.8	36.6	2.50 (1.15-5.73)	0.013	binned	20.3	36.6	2.27 (1.05-5.08)	0.024	binned	37.5	51.8	1.79 (0.91-3.53)	0.068
B	35:01	12.5	8.0	0.61 (0.20-1.94)	0.335	35:01:01	12.5	8.0	0.61 (0.20-1.94)	0.335					
B	40:02	14.1	7.1	0.47 (0.15-1.47)	0.135	40:02:01	14.1	7.1	0.47 (0.15-1.47)	0.135	40:02:01:01	10.9	6.3	0.54 (0.15-1.92)	0.269
B	51:01	6.3	11.6	1.97 (0.57-8.65)	0.247	51:01:01	6.3	11.6	1.97 (0.57-8.65)	0.247					
**B**	**52:01**	**20.3**	**8.9**	**0.38 (0.14-1.03)**	**0.031**	**52:01:01**	**20.3**	**8.9**	**0.38 (0.14-1.03)**	**0.031**	**52:01:01:02**	**20.3**	**8.9**	**0.38 (0.14-1.03)**	**0.031**
B	binned	46.9	64.3	2.04 (1.04-4.00)	0.024	binned	46.9	64.3	2.04 (1.04-4.00)	0.024	binned	68.8	84.8	2.54 (1.13-5.69)	0.012
DRB1	04:05	7.8	11.6	1.55 (0.49-5.82)	0.424	04:05:01	7.8	11.6	1.55 (0.49-5.82)	0.424	04:05:01:01	7.8	11.6	1.55 (0.49-5.82)	0.424
DRB1	09:01	18.8	8.9	0.42 (0.15-1.16)	0.058	09:01:02	18.8	8.9	0.42 (0.15-1.16)	0.058	09:01:02:01	18.8	8.9	0.42 (0.15-1.16)	0.058
DRB1	15:01	12.5	8.9	0.69 (0.23-2.13)	0.452	15:01:01	12.5	8.9	0.69 (0.23-2.13)	0.452	15:01:01:01	10.9	8.9	0.80 (0.26-2.62)	0.664
DRB1	15:02	15.6	7.1	0.42 (0.13-1.25)	0.074	15:02:01	15.6	7.1	0.42 (0.13-1.25)	0.074	15:02:01:01	15.6	7.1	0.42 (0.13-1.25)	0.074
DRB1	binned	45.3	63.4	2.09 (1.07-4.09)	0.020	binned	45.3	63.4	2.09 (1.07-4.09)	0.020	binned	46.9	63.4	1.96 (1.00-3.84)	0.033
DQA1	01:02	15.6	13.4	0.84 (0.33-2.23)	0.683	01:02:01	14.1	13.4	0.95 (0.36-2.62)	0.901	01:02:01:01	12.5	8.0	0.61 (0.20-1.94)	0.335
DQA1	01:03	21.9	16.1	0.68 (0.29-1.62)	0.337	01:03:01	21.9	16.1	0.68 (0.29-1.62)	0.337	01:03:01:01	15.6	8.9	0.53 (0.19-1.52)	0.178
DQA1	01:04	6.3	8.9	1.47 (0.4-6.69)	0.528	01:04:01	6.3	8.9	1.47 (0.40-6.69)	0.528	01:04:01:01	6.3	8.9	1.47 (0.40-6.69)	0.528
DQA1	03:01	10.9	13.4	1.26 (0.45-3.87)	0.636	03:01:01	10.9	13.4	1.26 (0.45-3.87)	0.636	03:01:01:01	10.9	13.4	1.26 (0.45-3.87)	0.636
**DQA1**	**03:02**	**21.9**	**9.8**	**0.39 (0.15-1.00)**	**0.028**	**03:02:01**	**21.9**	**9.8**	**0.39 (0.15-1.00)**	**0.028**	**03:02:01:01**	**21.9**	**8.9**	**0.35 (0.13-0.92)**	**0.016**
DQA1	03:03	7.8	16.1	2.26 (0.75-8.17)	0.118	03:03:01	7.8	16.1	2.26 (0.75-8.17)	0.118	03:03:01:03	6.3	11.6	1.97 (0.57-8.65)	0.247
DQA1	binned	15.6	22.3	1.55 (0.66-3.91)	0.284	binned	17.2	22.3	1.38 (0.60-3.38)	0.417	binned	26.6	40.2	1.86 (0.91-3.89)	0.069
DQB1	03:01	7.8	13.4	1.82 (0.59-6.73)	0.262	03:01:01	7.8	13.4	1.82 (0.59-6.73)	0.262					
DQB1	03:02	10.9	13.4	1.26 (0.45-3.87)	0.636	03:02:01	10.9	13.4	1.26 (0.45-3.87)	0.636	03:02:01:01	10.9	13.4	1.26 (0.45-3.87)	0.636
DQB1	03:03	20.3	9.8	0.43 (0.16-1.12)	0.051	03:03:02	20.3	9.8	0.43 (0.16-1.12)	0.051	03:03:02:02	20.3	9.8	0.43 (0.16-1.12)	0.051
DQB1	04:01	6.3	11.6	1.97 (0.57-8.65)	0.247	04:01:01	6.3	11.6	1.97 (0.57-8.65)	0.247	04:01:01:01	6.3	11.6	1.97 (0.57-8.65)	0.247
DQB1	06:01	21.9	15.2	0.64 (0.27-1.53)	0.262	06:01:01	21.9	15.2	0.64 (0.27-1.53)	0.262	06:01:01:01	21.9	15.2	0.64 (0.27-1.53)	0.262
DQB1	06:02	12.5	8.0	0.61 (0.20-1.94)	0.335	06:02:01	12.5	8.0	0.61 (0.20-1.94)	0.335	06:02:01:01	12.5	8.0	0.61 (0.20-1.94)	0.335
DQB1	binned	20.3	28.6	1.57 (0.72-3.57)	0.227	binned	20.3	28.6	1.57 (0.72-3.57)	0.227	binned	28.1	42.0	1.85 (0.91-3.82)	0.067
DPA1	01:03	40.6	43.8	1.14 (0.58-2.23)	0.687	01:03:01	40.6	43.8	1.14 (0.58-2.23)	0.687	01:03:01:01	32.8	25.0	0.68 (0.33-1.43)	0.266
DPA1											01:03:01:05	4.7	14.3	3.39 (0.91-18.79)	0.048
DPA1	02:01	21.9	14.3	0.60 (0.25-1.44)	0.198	02:01:01	21.9	14.3	0.60 (0.25-1.44)	0.198	02:01:01:02	14.1	12.5	0.87 (0.33-2.45)	0.767
DPA1	02:02	37.5	42.0	1.21 (0.61-2.39)	0.561	02:02:02	37.5	42.0	1.21 (0.61-2.39)	0.561	02:02:02:01	37.5	42.0	1.21 (0.61-2.39)	0.561
DPA1											binned	10.9	6.3	0.54 (0.15-1.92)	0.269
**DPB1**	**02:01**	**32.8**	**17.9**	**0.45 (0.21-0.97)**	**0.024**	**02:01:02**	**32.8**	**17.9**	**0.45 (0.21-0.97)**	**0.024**	**02:01:02:01**	**25.0**	**15.2**	**0.54 (0.23-1.25)**	**0.108**
DPB1	04:02	4.7	14.3	3.39 (0.91-18.79)	0.048	04:02:01	4.7	14.3	3.39 (0.91-18.79)	0.048	04:02:01:02	4.7	13.4	3.14 (0.84-17.54)	0.067
DPB1	05:01	32.8	39.3	1.32 (0.66-2.68)	0.392	05:01:01	32.8	39.3	1.32 (0.66-2.68)	0.392	05:01:01:01	29.7	31.3	1.08 (0.53-2.24)	0.829
DPB1	09:01	9.4	10.7	1.16 (0.38-3.97)	0.778	09:01:01	9.4	10.7	1.16 (0.38-3.97)	0.778	09:01:01	9.4	10.7	1.16 (0.38-3.97)	0.778
DPB1	binned	20.3	17.9	0.85 (0.37-2.03)	0.688	binned	20.3	17.9	0.85 (0.37-2.03)	0.688	binned	31.3	29.5	0.92 (0.45-1.91)	0.804
